# Impact of floods on undernutrition among children under five years of age in low- and middle-income countries: a systematic review

**DOI:** 10.1186/s12940-022-00910-7

**Published:** 2022-10-24

**Authors:** Caroline Noel Agabiirwe, Peter Dambach, Thabile Constance Methula, Revati K Phalkey

**Affiliations:** 1 Division of Epidemiology and Public Health, School of Medicine, University of Nottingham, Nottingham, UK; 2 Heidelberg Institute of Global Health, University of Heidelberg, Heidelberg, Germany

**Keywords:** Flood, Climate change, Undernutrition, Nutrition, Stunting, Wasting, Underweight, Micronutrient deficiency

## Abstract

**Background:**

Weather and climate-related disasters, including floods, impact undernutrition through multiple pathways, including food security, inadequate child care practices, and water and sanitation. This review aimed to provide systematic evidence of the impact of floods on undernutrition in children under five years of age in Low and Middle-income countries (LMICs).

**Methods:**

We searched PubMed, Web of Science, Embase, MEDLINE, CINAHL and Scopus for peer-reviewed articles. Popline, WHO Library database (WHOLIS), the International Disaster database (EM-DAT), Food and Agriculture Organisation (FAO), UNICEF and Eldis were searched for grey literature articles. Database searches were first conducted in 2016 and updated in 2020. We included English language articles that reported the effect of floods on undernutrition outcomes in children under 5 years of age in LMICs, without limitation to study design and year of publication. The quality of selected studies was assessed using the National Institutes of Health (NIH) tool for Observational Cohort and Cross-Sectional Studies.

**Results:**

Of the 5701 articles identified, 14 met our inclusion criteria. The review noted stunting as the most frequently reported significant form of undernutrition in flood-affected areas. Severe and recurrent floods showed the greatest impact on undernutrition. Due to weak and limited evidence, the study is inconclusive on the most significant forms within the short-term and intermediate periods following floods. On the other hand, stunting was noted as the most frequently reported significant form of undernutrition in the long-term period following floods. There was generally little evidence of the effect of floods on micronutrient deficiencies. Factors associated with child undernutrition in the flood-affected areas included age, gender, diarrhoea, maternal and paternal education, maternal age, household size, land ownership and socioeconomic status. Overall, the quality of the evidence was fairly weak, with the main challenge lying in the inability of the studies to establish causal pathways for the observed effects.

**Conclusions:**

The review suggests clear plans and strategies for preventing and reducing the long-term impact of floods on undernutrition in children under five years. Future research utilising long-term prospective data is indispensable to provide more robust evidence to guide better prevention measures, response decisions and interventions.

**Supplementary Information:**

The online version contains supplementary material available at 10.1186/s12940-022-00910-7.

## Background

Malnutrition is a global public health problem and one of the target areas for attaining the Sustainable Development Goals (SDGs) [1]. The second SDG aims to end malnutrition in all its forms [1]. Malnutrition affects all age groups; however, infants and young children are the most at risk due to their high nutritional requirements for growth and development [2]. Malnutrition is a term that covers both over-nutrition and undernutrition. The focus of this review is undernutrition, an outcome of insufficient food intake and repeated infectious diseases, among other factors [3]. Globally, of the 6.9 million deaths of children under the age of 5 years, 3.1 million deaths are attributed to malnutrition, among them being undernutrition [4]. According to the 2017 joint global malnutrition estimates, two-thirds (66%) of all stunted children and a quarter (25%) of all wasted children under five years lived in lower-middle-income countries [5]. Internationally recommended indicators of child malnutrition are a combination of body measurements (weight or height) and age [2]. The combinations result in three main indicators; Height-for-Age (HFA), Weight-for-Height (WFH) and Weight-for-Age (WFA), used for assessing common forms of undernutrition that are stunting, wasting and underweight, respectively [6]. Stunting is defined as HFA ≤ -2 Standard deviations (SD) of the World Health Organization (WHO) Child growth standards median; Wasting as WFH ≤ -2SD and Underweight as WFA ≤ -2SD of the WHO Child growth standards median [7]. Another form of undernutrition among infants and children is micronutrient deficiencies well-defined as the lack of essential vitamins and minerals required in small amounts by the body for proper growth and development, including but not limited to: iron, zinc, calcium, iodine, vitamin A and C [8]. Micronutrient deficiencies account for close to 7.3% of the global disease burden, with an estimated 12% of deaths among children under five years of age attributed to the lack of the four most common micronutrients, including vitamin A, iron, iodine and zinc deficiencies [4]. Flood is a general term for the overflow of water from a stream channel to normally dry land in the flood plain (riverine flooding), to higher than normal levels along the coast and in lakes or reservoirs (coastal flooding), as well as pending water at or near the point where the rain fell (flash floods) [9]. A systematic review conducted in 2013 reported that in the past 30 years, approximately 2.8 billion people were affected by floods, 4.5 million were left homeless, and an estimated 540,000 were killed [10]. In 2019, floods represented the highest occurrence of all disaster types and accounted for 43.5% of all deaths due to disasters [11]. Globally, although disastrous floods occur in other continents, Asia and Africa seem to be the most affected. The Intergovernmental Panel on Climate Change (IPCC) has reported that, based on available scientific literature, disasters, including floods, have been attributed to climate change leading to disruption of water and food supply, damaging infrastructure and settlements, as well as increasing morbidity and mortality [12]. In Bangladesh, excessive flooding, partly due to changes in global sea surface temperatures (such as those induced by El Niño), affected rice production, prices, and child nutrition [13, 14]. Effects of flooding relate to immediate, underlying and basic causes of child undernutrition with reference to the UNICEF conceptual framework of malnutrition [15]. Weather and climate-related disasters, including floods, impact undernutrition through multiple pathways, including food security, inadequate child care practices, and water and sanitation [6]. According to the WHO, floods may trigger food insecurity, increase malnutrition and enhance vulnerability to infectious diseases [16].

There is some (medium) evidence of the increase in flooding due to projected heavy rainfall in some areas across the globe [17]. In addition, the expected wetter conditions and flooding resulting from weather and climate change are more likely to increase food insecurity and undernutrition [16]. However, there remains a noticeable gap in the presence of solid evidence of the impacts of floods on undernutrition in the forms of stunting, wasting, underweight, and micronutrient deficiencies, especially among children under five years of age. A previous systematic review focused on assessing the impact of general weather and climate change variables on undernutrition, mainly focusing on stunting [18]. A more recent one focused on the assessment of the effects of droughts, floods and fluctuations in temperature and precipitation on wasting, underweight and stunting among children and adult populations [19]. In view of efforts to reduce child undernutrition in all its forms (i.e., wasting, underweight, stunting and micronutrient deficiencies) in flood-affected areas, there is an indispensable need to generate robust evidence that will guide future interventions, appropriate preparedness and preventive measures in the period following floods. Therefore, objectives of this review are to systematically synthesise evidence of the impact of floods on wasting, stunting, and underweight and micronutrient deficiencies in the short-term, intermediate and long-term periods following the floods and to identify factors associated with undernutrition among children under the age of five years in flood-affected areas. The overall aim of the review is to provide systematic evidence of the impacts of floods on undernutrition in children under five years of age in LMICs.

## Methods

### Study inclusion and exclusion criteria

We included studies that reported undernutrition outcomes due to flood exposure in LMICs as defined by the World Bank in 2015 (i.e., countries with gross national income per capita ≤ 12,735 USD) [20]. Eligibility of studies was assessed following the “Population”, “Exposure”, “Comparator”, “Outcome” (PECO) statement below [21]:

- Population: children under 5 years of age in LMICs
- Exposure: flooding event(s) of any type, cause and intensity
- Comparison: where available, population not exposed to a flooding event(s) of any type, cause and intensity
- Outcome: the four forms of undernutrition, i.e., stunting, wasting, underweight and micronutrient deficiencies

We included English language articles from LMICs, with no restrictions to study design, year of publication and duration of follow-up post flood exposure. We excluded any study that assessed the impact of floods on undernutrition in high-income countries, focused on children above five years of age, had no full-text article (i.e., only abstracts available), were editorial materials, proceeding papers, conference papers and short communications.

### Literature sources and search terms

The following electronic databases were searched for peer-reviewed articles; PubMed, Web of Science, Embase, MEDLINE, CINAHL and Scopus. Popline, WHO Library database (WHOLIS), the International Disaster database (EM-DAT), Food and Agriculture Organisation (FAO), UNICEF and Eldis were searched for grey literature articles. To broaden the scope of the review, there were no restrictions to study design and year of publication, and reference lists of selected articles were thoroughly searched to identify relevant additional articles. A scoping search in PubMed enabled the identification of key terms for the search strategy. The initial search strategy involved a combination of applicable key search terms under the two key main concepts: floods and undernutrition. The identified search terms were then modified for other databases considering controlled vocabulary where necessary. Key terms were searched as MeSH terms, free texts, phrases, subject terms and fielded search (topic, title, abstract and keywords). The Boolean operator “OR” was used to combine terms of the same concept, while “AND” was used to combine terms from the two concepts. Two researchers independently conducted the searches, with the first search conducted in March and April 2016, the second in October and November 2016 and an update in July and August 2020.

### Study selection, data extraction and synthesis

All studies obtained were uploaded into Endnote X7, and duplicate articles were deleted automatically. The remaining articles were screened independently based on the inclusion and exclusion criteria. Three main steps were followed for the screening process: title, abstract and full-text screening. At the full-text screening stage, codes representing reasons for inclusion or exclusion were assigned to each of the remaining articles. Double screening of all full text articles was conducted to obtain final articles for the review. A flow diagram was used to illustrate the various stages of study selection, the final number of full-text articles obtained and the reasons for exclusion of articles at the full-text screening stage. The 2009 Preferred Reporting Items for Systematic Reviews and Meta-analysis (PRISMA) statement was adhered to during the entire review process [22]. Data from all the studies obtained after screening was summarised in an Excel data extraction form, including study characteristics, the year the flood occurred, duration of follow-up post flood exposure, outcomes assessed, significant outcomes, factors associated with undernutrition outcomes and main findings (see Tables 1 and 2). Data analysis was carried out descriptively, and a narrative of results from the various studies was provided under two main sections, i.e. 1) impact of floods on undernutrition and 2) factors associated with undernutrition in flood- affected areas. Results were also presented in terms of time point period post flood (i.e., time after floods when study was conducted or outcome measured) as short term (within 1–4 months), intermediate (six months) and long term (one year) periods in relation to the reported significant form of child undernutrition.

**Table 1:** Study Characteristics and summary of findings

Author, year	Country	Study Design and Type of data	Sample size	Year data collected	Study Setting	Age of children(months)	Time flood occurred	Duration after the flood when the study was conducted	Exposure measure	Outcome assessed	Significant outcome due to flood exposure	Summary of findings
Stewart et al., 1990 [23]	Bangladesh	Cross-sectional study, Primary	281(Oct), 264(Dec)	October& Dec 1988	Rural	6–35	September 1988	one month (Oct); 3 months (Dec) post-flood	None reported	Stunting, wasting & underweight	None reported	No significant difference in mean % NCHS median for wasting, underweight and stunting between October and December (3 months after floods)
Choudhury and Bhuiya, 1993 [24]	Bangladesh	Cross-sectional-Household based nutrition survey, Primary	906 pre-flood & 888 post-flood	Pre-flood March–April 1987 and Post-flood June-July 1988	Rural	0–23	July to December 1987	6 months	Pre-flood and post-flood exposure in severely exposed and moderately exposed areas	Underweight	Underweight	Post-flood underweight increased to 11% after the flood compared to 5% before the flood (*p* < 0.001). The proportions of severely malnourished children in less flood-affected villages during the pre-flood and post-flood periods remained more or less constant at 12% and 11%, respectively, and were insignificant. The impact of floods on underweight was statistically significant (β = 0.48 *p* < 0.05)
del Ninno C et al., 2001 [25]^a^	Bangladesh	Cross-sectional study-Household survey, Secondary	384	Between November 1998 and December 1999	Rural	0–59	July to September 1998	2 months	Flood exposure index (categories, i.e., moderate, severe & very severe)	Stunting & wasting	Stunting & wasting	After the floods, 24% of pre-school children (0-5 years) were wasted while 55% were stunted. A child living in a household that was severely exposed to the floods had seven times (OR = 7.30) increased risk of being wasted than one not exposed. The increased risk was almost five times (OR = 4.87 for households that were very severely exposed to floods. Floods also led to a significant increase in stunting (OR = 2.18) for children living in very severely exposed households
Hossain & Kolsteren, 2003 [26]	Bangladesh	Cross-sectional study, Secondary	180 common to both surveys (August and December)	August &December 1998	Rural	6–59	July to October 1998	4 months	None reported	Wasting	Wasting	8% of those who were normal during the floods became malnourished 4 months later. In addition, 4% of those who were malnourished during the floods, remained malnourished after the floods. There was a significant decline in the prevalence of wasting, i.e., from 17% in August to 12% in December(*p* = 0.011)
del Ninno & Lundberg, 2005 [27]	Bangladesh	Cross-sectional study Household based survey, Secondary	237(Measured in all the 3 rounds)	Between November 1998 and December 1999	Rural	0–59	July to September 1998	2 months, 6 months (April 1999) & 15 months post-flood	Household flood exposure index (categories i.e. not exposed, moderate, severe & very severe)	Stunting and wasting	Stunting	There was a significant difference in HFA mean z-scores (*p* < 0.05) for children older than one year from flooded compared to non-flooded areas. No significant difference in wasting for flooded compared to non-flooded
Goudet et al., 2011 [28]	Bangladesh	Cross-sectional study-Household based survey, Secondary	143	Between November 1998 and December 1999	Rural	12–36	July to September 1998	2 months (baseline) and 15 months	Household flood exposure index (categories, i.e., not exposed, moderate, severe & very severe)	Stunting, wasting and underweight	stunting & wasting	Severe and moderate levels of flood exposure were observed as predictors of stunting (OR = 8.210, 95% CI: 1.194–56.464, significant at 5% level) and wasting (OR = 25.06, 95% CI: 1.81–347.45, significant at 5% level), respectively. For underweight children, there were no significant effects observed
Rodriguez-Llanes et al., 2011 [14]	India	Cross-sectional study-community based, Primary	352	Sep-09	Rural	6–59	September 2008 (similarly flooded in August 2006)	one month	Level of children's exposure to floods, i.e., flooded vs non-flooded	Stunting, wasting and underweight	stunting & underweight	The prevalence of stunting was 38.7% in the flooded compared to the non-flooded areas (23.0%). The prevalence of underweight was higher in children living in flooded areas (20.9%) compared to those from non-flooded areas (13.1%). The prevalence of wasting was similar in children in the flooded areas compared to those in the non-flooded with 12.2% and 11.9%, respectively. Children living in flooded communities were more likely to be stunted (Adjusted PR = 1.60, 95% CI:1.05–2.44) and underweight (Adjusted PR = 1.86 95% CI: 1.04–3.30) but not wasted (Adjusted PR = 1.21, 95% CI: 0.61–2.42) relative to those in the non-flooded villages
Hossain et al., 2013 [29]	Pakistan	Cross-sectional study, Primary	2819	Oct-Dec 2010	Rural & Urban	6–59	July and August 2010	2 months	Not stated	Stunting, wasting, underweight and Micronutrient deficiencies (Vitamin A, D and iron deficiency anaemia)	None reported	Wasting was higher in severely flooded areas (21–23%) compared to moderately flooded areas (14%). Underweight in severely flooded areas was higher (46–48%) compared to less affected areas (39–40%). The prevalence of stunting was higher in severely flooded areas (52–54%) compared to moderately flooded areas (47–53%). In the other study area that was severely flooded, 16% of the children were wasted, 48% were stunted, 65% were underweight, 55% were Vitamin A deficient, 58% were Vitamin D deficient, and 78% were anaemic
Quddus and Bauer, 2013 [30]	Bangladesh	Cross-sectional study-community based, Primary	156	January-June 2009	Rural	24–59	Not stated	Not stated	Not stated	Stunting, wasting and underweight	None reported	In the river-flooded communities, the prevalence of underweight was higher (70.2%), followed by wasting (56%) and stunting (40.4%). However, the nutrition indicators’ prevalence was lower in river-flooded as compared to the non-flooded areas (Hills/forest and coastal areas)
Islam et al., 2014 [31]	India	Cross-sectional study-community based, Primary	500	2011–2012	Rural	0–59	Not stated	Not stated	Not stated	Stunting, wasting and underweight	None reported	The prevalence of stunting was higher (30.4%), followed by underweight (29%) and wasting (21.6%). The prevalence of wasting in the flood-affected areas was higher (21.6%) compared to the non-flood-affected areas (13.7%)
Rodriguez-Llanes et al., 2016a [32]	India	Cross-sectional survey population-based, Primary	879	September-09	Rural	6–59	September 2008 (similarly flooded in August 2006)	One year	Flood villages vs non-flooded	Stunting, wasting and underweight	Wasting & underweight	The prevalence of wasting among children in areas flooded in 2006 and 2008 was 51.6%, 41.4% in those flooded only in 2008, and 21.2% in children inhabiting non-flooded communities. The increased prevalence of wasting and underweight for children in flooded compared to non-flooded communities was significant, while stunting was not significant. The Adjusted Prevalence Ratio (APR) of wasting in flooded communities relative to the non-flooded was 2.30 95% CI 1.86–2.85 for those flooded twice (2006 and 2008) and 1.94, 95% CI: 1.43–2.63) for those flooded once in 2008. Children additionally exposed to floods in 2008 had more than three times higher prevalence of severe wasting relative to those from the non-flooded areas (APR: 3.37, 95% CI: 2.34–4.86), and this effect nearly doubled that of those only exposed to 2008 floods. The adjusted Prevalence(APR) of underweight in flooded communities relative to the non-flooded was 1.48, 95% CI: 1.21–1.81 for those flooded twice (2006 and 2008) and 1.53, 95% CI: 1.09–2.13 for those flooded once in 2008
Rodriguez-Llanes et al., 2016b [33]	India	Cross-sectional survey population-based, Primary	879	September 2009	Rural	6–59	September 2008 (similarly flooded in August 2006)	One year	Exposure to floods, i.e., flooded vs non-flooded	Stunting and wasting	None reported	The prevalence of stunting was relatively similar in flooded (30.4%) and non-flooded areas (29.0%). Prevalence of wasting was higher in flooded (51.5%) as compared to non-flooded areas (20.3%
Gaire et al., 2016 [34]	Nepal	Cross-sectional survey population-based, Secondary	2,111	Between February and June 2011	Rural & Urban	6–59	2007–2010	1-4yrs	Flood vs non-flooded	Stunting	Stunting	Floods significantly impacted severe and moderate stunting i.e., adjusted OR = 0.57, 95% CI: 0.31, 0.96 and adjusted OR = 0.66, 95% CI: 0.41, 0.94 respectively
Dimitrova and Bora, 2020 [35]	India	Cross-sectional survey population-based, Secondary	256,244	2015–2016	Rural &Urban	0–59	2009–2016	1-6 years	SPEI index (Standardized precipitation and evapotranspiration) > = 2 categorised as flood events	Stunting and wasting	Stunting	Exposure to monsoon season floods in infancy increased the risk of severe stunting by 4%(OR = 1.040, 95% CI: 1.011–1.070)

^a^No confidence intervals were provided; the study only reported regression coefficients significant at 10% or less

**Table 2:** Summary of main factors associated with undernutrition

Author, year	Country	Demographic factors	Maternal factors	Paternal factors	Child-health related factors	Infant and Young Child Feeding practices	Household factors	Socioeconomic factors	Food security and livelihood factors
Stewart et al., 1990 [23]	Bangladesh	√	√		√			√	
Choudhury and Bhuiya, 1993 [24]	Bangladesh	√	√		√			√	
del Ninno C et al., 2001 [25]	Bangladesh	√	√	√				√	√
Hossain & Kolsteren, 2003 [26]	Bangladesh				√				
del Ninno & Lundberg, 2005 [27]	Bangladesh	√							
Goudet et al., 2011 [28]	Bangladesh	√	√		√		√		
Rodriguez-Llanes et al., 2011 [14]	India	√					√		
Hossain et al., 2013 [29]	Pakistan	√							
Quddus and Bauer, 2013 [30]	Bangladesh								
Islam et al., 2014 [31]	India	√	√	√	√	√	√	√	
Rodriguez-Llanes et al., 2016a [32]	India	√	√				√		
Rodriguez-Llanes et al., 2016b [33]	India		√	√			√	√	√
Gaire et al., 2016 [34]	Nepal	√	√		√			√	
Dimitrova and Bora, 2020 [35]	India	√	√				√	√	
Total		11	9	3	6	1	6	7	2

### Quality assessment of selected studies

We assessed each of the selected study’s quality using the NIH tool for Observational Cohort and Cross-Sectional Studies [36]. Each study was evaluated against the 14 criteria questions in the assessment tool and rated as “Good”, “Fair”, or “Poor” based on the level of risk of bias. Overall, studies with a low risk of bias were rated as “Good”, whereas those with a high risk of bias were rated as “Poor”. Two independent reviewers (CNA and PD) assessed the quality of the studies.

## Results

### Study selection and characteristics

**Fig. 1:**
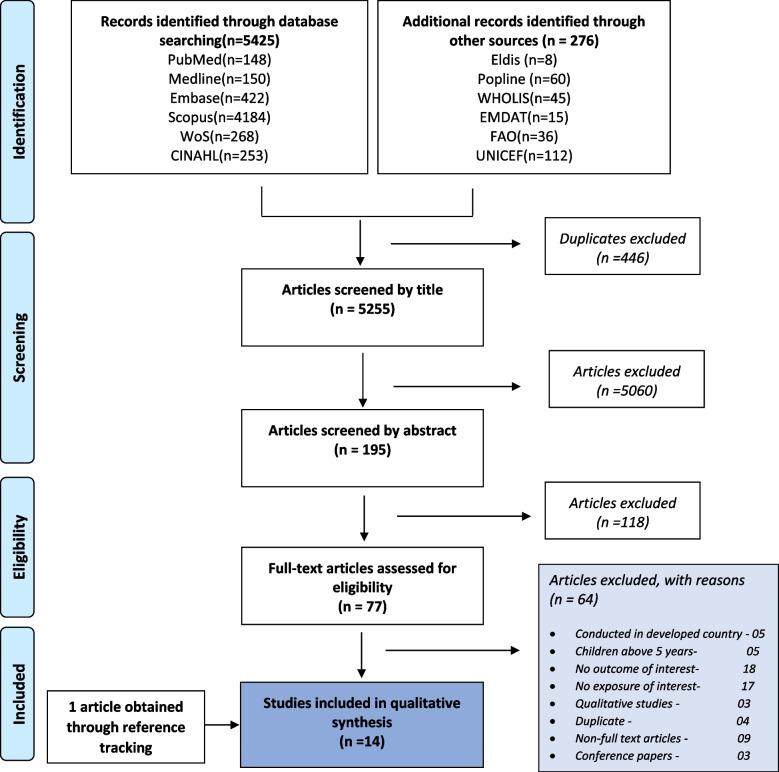
Flow diagram of inclusion and exclusion of studies

The search strategy yielded a total of 5701 articles, and of these 446 duplicates were deleted. The remaining 5255 articles were screened for title and abstract, 77 full-text articles were assessed for eligibility, and fourteen (14) articles were included in the review, including one obtained through reference tracking. Figure 1 illustrates a summary of the study selection process.

All the studies were from South Asia (Bangladesh, India, Nepal and Pakistan), of cross-sectional design, published between 1990 and 2020. The sample size of the studies varied greatly, ranging from 143 to 256,244 children. The age group of children below five years varied and ranged from 0–59 months. The most commonly assessed age group was 6–59 months, assessed in six of the 14 studies. Eleven of the 14 studies reported exposure to seasonal floods (during the monsoon season) that were sometimes recurrent, severe and/or lasting approximately three to four months. Duration of data collection post-flood exposure varied, with five studies reporting a period of up to four months, one study reporting six months and six studies at or after one year. Of the 14 studies, 12 assessed the impact of floods on wasting and stunting, 8 on underweight and 2 on micronutrient deficiencies. Seven of the 14 studies used primary data, while the other seven utilised existing data (secondary data), all from cross-sectional surveys. A majority of the studies (11) were conducted in rural areas, while 3 were in rural and urban settings. Eight studies reported using the 2006 WHO Child growth reference standards [37], while six used the National Center for Health Statistics (NCHS) median reference standards for the classification of nutrition status.

### Quality assessment of included studies

Overall, the quality of the studies varied, with three of the 14 studies rated as “Good”, seven rated as “Fair”, and four as “Poor” (see Additional file 1). The main weakness in the studies was the lack of sufficient time between exposure and outcome to allow for an association to be observed, as noted in 4 studies [14, 23, 25, 29] rated as “Fair” or “Poor”. Other reasons for rating studies as “Fair” or “Poor” included the use of unreliable outcome assessment tools (e.g., the NCHS median reference standard) [24, 27, 28], the lack of time separating exposure and outcome measurements [30, 31, 38], the use of unreliable outcome data [34], and selection bias [26].

### Impact of floods on undernutrition

Generally, stunting was the most frequently reported form of undernutrition in areas affected by floods. Nine of the 14 studies reported significant undernutrition outcomes due to flood exposure. Six of the 9 studies reported significant effects on stunting, 4 on wasting and 3 on underweight.

#### Floods and undernutrition

##### Seasonally and/or recurrently flooded areas

Eight of the 11 studies conducted in seasonally and/or recurrently flooded areas, mainly in India and Bangladesh, reported significant effects on undernutrition outcomes. Of these 8 studies, 5 reported significant effects on stunting [14, 25, 27, 28, 35], 4 on wasting [25, 26, 28, 32] and 3 on underweight [14, 24, 32]. Two of the 7 studies were rated as “Good” (i.e. having a low risk of bias) [32, 35], 5 as “Fair” [14, 24, 25, 27, 28] and one [26] as “Poor” (i.e. having a high risk of bias).

In India, a study conducted one month after the floods [14] reported that children living in repeatedly flooded communities were more likely to be underweight (Adjusted prevalence rate (APR) = 1.86, 95% CI: 1.04–3.30) and stunted (APR = 1.60, 95% CI: 1.05–2.44) but not wasted (APR = 1.2, 95% CI: 0.61–2.42) relative to those in the non-flooded villages. In the one-year follow-up study (rated as “Good”) [32], a significant increase in the prevalence of underweight (APR = 1.48, 95% CI: 1.21–1.81) and wasting (APR = 2.30, 95% CI: 1.86–2.85) were reported but not stunting, for communities that were flooded twice (2006 and 2008) relative to the non-flooded communities. A more recent study (rated as “Good”) reported an increased risk of stunting (OR = 1.040, 95% CI: 1.011–1.070) for children exposed to seasonal (monsoon) floods in infancy [35].

In Bangladesh, a study conducted two months after the floods (rated as “Fair”) observed a two-fold (OR = 2.18) increase in stunting for children living in villages very severely exposed [25]. The study also noted a seven-times (OR = 7.30) increased risk of wasting for a child in a household severely exposed to floods and a nearly five times higher risk (OR = 4.87) for those living in households very severely exposed to floods (no confidence intervals were provided for the reported ORs, the study only reported significant regression coefficients at 10% level) [25]. A follow-up study, a year later, reported a significant difference in HFA mean z-scores (about 0.5 of one SD) for children from flooded compared to non-flooded areas [27]. In another follow-up study conducted 15 months after the floods (i.e., a year after a baseline of 2 months post the floods), severe and moderate levels of flood exposure were observed as predictors of stunting (OR = 8.210, 95% CI: 1.194–56.464) and wasting (OR = 25.06, 95% CI: 1.81–347.45), respectively [28]. The only study conducted 6 months post floods reported a significant increase in the prevalence of underweight compared to that before the floods (*p* < 0.001) [24]. On the contrary, Hossain & Kolsteren (rated as “Poor”) observed a significant decline in the prevalence of wasting in the recovery period (4 months post-floods) [26].

##### Other flood-affected areas

Three of the 14 studies were conducted in flood-affected areas other than seasonally and/or recurrently flooded in Bangladesh, India and Nepal. Two of the 3 studies were rated as “Poor” quality [30, 31] (i.e., having a high risk of bias) and one as “Fair” [34]. In Bangladesh, the prevalence of underweight in the river-flooded communities was higher (70.2%), followed by wasting (56%) and stunting (40.4%) [30]. In these areas, the prevalence of all undernutrition outcomes was lower compared to the non-flooded areas (i.e., hills/forest and coastal areas). However, the differences between the areas were not statistically significant. In India, although with no significant values reported, the prevalence of wasting in the river flooded areas was noted as higher (21.6%) than that in non-flood affected areas (13.7%) [31].

Contrary to findings on the effects of floods on stunting from the other studies, in Nepal [34], exposure to floods showed protective effects on stunting (adjusted OR = 0.42, 95% CI: 0.26—0.67).

#### Follow-up period post flood exposure and undernutrition outcomes

##### Short-term and intermediate periods

Five [14, 23, 25, 26, 29] of the 14 studies assessed the effect of floods on undernutrition within the four months post-flood exposure (short-term). Three [14, 25, 29] of the five studies were rated as “Fair” quality and two [23, 26] as “Poor” quality. Two of the five studies reported significant effects of floods on stunting in the short-term period [14, 25]. The studies also reported increased wasting [25] and underweight [14]. However, inconsistent findings were reported by other studies from India [14], Pakistan [29] and Bangladesh [26]. The study in India [14] reported a higher prevalence of wasting in the flooded areas compared to the non-flooded one-month post-exposure, but the effect was not statistically significant. In Pakistan, the prevalence of stunting and underweight was higher in the severely flooded regions compared to the less affected regions two months after the floods [29]. However, the differences between the regions were not significant. In Bangladesh, a significant decline in the prevalence of wasting was observed four months after the floods (*p* = 0.011) [26].

Only one study assessed the nutrition status of children at six months (intermediate period) post flooding. The study (rated as “Fair”) reported a significant increase in underweight in the flood-affected areas from 5 to 11% (*p* < 0.001) [24].

##### Long-term period

Six of the 14 studies assessed the effect of floods on undernutrition at or after the one year following floods [27, 28, 32–35]. Three [32, 33, 35] of the six studies were rated as “Good” quality and the other three [27, 28, 34] as “Fair”. Stunting was the most frequently reported significant form of undernutrition. Of the six studies conducted after one-year following floods, 4 reported significant effects on stunting [27, 28, 34, 35]. One of the 4 studies rated as “Good” quality [35] reported small but significant effects on stunting (OR = 1.040, 95% CI: 1.011–1.070) for children exposed to floods in infancy [35]. Contrary to findings from the four studies, a reduced risk of severe and moderate stunting was observed in flooded compared to non-flooded areas [34].

One of the 6 studies conducted at or after the one-year follow-up period reported significant effects on underweight [32]. The study observed a statistically significant increase in the prevalence of underweight (Adjusted prevalence ratio (APR) = 1.53, 95% CI: 1.09–2.13) among children from flooded villages compared to the non-flooded.

One study reported a significant increase in the prevalence of wasting (APR = 1.94 95% CI: 1.43- 2.63) a year later for children living in villages flooded in 2008 compared to the non-flooded villages [32]. Another study reported a higher prevalence of wasting in flooded (51%) as compared to non-flooded areas (20.3%) [33].

### Factors associated with undernutrition outcomes

Factors significantly associated with undernutrition in flood-affected areas were grouped into eight categories (see Table 2). Overall demographic, child-health related, maternal, household and socioeconomic factors were the most frequently reported factors associated with child undernutrition.

#### Wasting

##### Demographic factors (Age and gender)

Four [23, 28, 31, 32] of the 14 studies assessed age as a factor related to wasting in flood affected areas. Generally, children aged below 24 months were reported as being more vulnerable to wasting due to flood exposure. In Bangladesh, significant wasting was reported for those aged 13–24 months [23], and another study reported a reduced risk of wasting for children aged 24–36 months as compared to those aged 12–23 months (*p* < 0.05) [28]. In India, the cohort of children younger than 12 months during the previous flood in 2006 (aged 36–47 months, one year after the 2008 floods) presented the most significant difference in the prevalence of wasting (APR: 4.01; 95% CI: 1.51- 10.63) compared to those of the same age and never affected by the floods [32]. On the contrary, a study in India reported a high prevalence of wasting in those aged 24–36 months in the flood affected areas as compared to the other age groups with statistically significant differences (*p* < 0.05) [31]. Male children in flood-affected areas were more vulnerable to wasting than females. Two [27, 31] of the three studies that assessed gender as a determinant of wasting in flood-affected areas reported males as more vulnerable to wasting than females.

##### Child health-related factors and Infant and young child feeding (IYCF) practices

Two studies [23, 26] identified diarrhoea as significantly associated with wasting following a flood. Only one study in India assessed the association between IYCF practices and undernutrition in flood-affected areas [31]. According to the survey, exclusive breastfeeding and the child’s age (months) at the introduction of complementary feeding were significantly associated with wasting. Exclusive breastfeeding was associated with a lower prevalence of wasting, while the delayed start of complementary feeds was associated with a higher prevalence of wasting.

##### Maternal and paternal factors (Education and age)

Overall, maternal and paternal education were associated with wasting in the long-term following floods, while in the short-term period, no significant effects were observed. Del Ninno and colleagues [25] observed no significant effect of paternal education on child wasting in the short-term (two months) following the floods. Three of the 4 studies [23, 25, 26] that assessed maternal education observed no significant effects on child wasting within the short-term period following floods. However, a study conducted a year following floods identified maternal education as a significant determinant of wasting [32]. Similarly, paternal education was noted as a factor associated with wasting a year following flood exposure, with two studies reporting a significant association [32, 33]. Maternal and paternal age were noted as protective factors against wasting. A study in India reported that maternal age at birth was associated with a reduction in child wasting [33], with each additional year reducing the prevalence of wasting by 3.4% (*p* = 0.005). A study in Bangladesh reported that increased paternal age was associated with a reduced risk of wasting for children living in flooded villages [25]. However, no effects were observed at the household level [25].

##### Household, socioeconomic, food security and livelihood factors

Household factors assessed included family size and the number of children under five years. Family size was associated with wasting, while no association was observed for the number of children under five years. In Bangladesh, wasting was not related to the number of children under five years (eating from the same kitchen) one month after the floods [14]. A study in India reported that wasting was significantly associated with family size (*p* = 0.0001), with the highest prevalence among those from larger families of 9–11 members [31]. Three studies assessing socio-economic factors (socioeconomic class, access to loans and land ownership) reported significant effects on wasting in the short-term following floods. One study reported socioeconomic class as a significant determinant of wasting [31]. In another study, access to loans significantly improved nutrition status four months after the floods [26]. Stewart et al. [23] observed differences in the mean WFH for different categories of land ownership (*p* = 0.02) three months following the floods, with significantly lower WFH mean for those with no land (landless) or less than 1.5 acres of land. On the contrary, Hossain & Kolsteren [26] reported that for families with no land owned, there was no significant difference in wasting between those whose nutrition status improved or declined four months after the floods [26]. In India, an increase in a hectare of land owned led to a decrease in the prevalence of wasting among children in flooded areas a year later, but the effect was not-significant (*p* = 0.56) [33]. Other significant determinants of wasting post floods included depending on own food stocks [26] and households’ dedication to agricultural livelihoods [33].

#### Stunting

##### Demographic factors (Child age and gender)

Eight of the 14 studies identified age as a factor related to stunting in flood-affected areas [14, 23, 25, 27–29, 31, 33]. Overall, children above 12 months were more likely to be stunted with studies observing significant effects in the short term [14, 25] and long-term periods following floods [27, 28]. There were variations in the age groups most affected i.e. those aged 12–23 months [28], 20–34 months [25], 26-36 months [14] and 48–59 months [27, 31]. Male children were at a higher risk of stunting than females. Two [23, 31]of the five studies that reported gender as a determinant of stunting noted significantly higher stunting levels among male children compared to females. Although with no significant effects reported, Hossain et al. [29] observed a higher prevalence of stunting among boys than girls in severely and moderately flood-affected areas.

##### Child health-related factors and IYCF practices

In Bangladesh, child health-associated vulnerabilities of stunting a year after the floods included the number of days of diarrhoea during the floods, number of days of Acute respiratory infection (ARI) during the floods and pre-existing undernutrition status (stunted) [28]. Only one study assessed the association between IYCF practices and stunting. The study reported that stunting levels were significantly higher among children that were not exclusively breastfed, not given colostrum, given prelacteal feed and those who had delayed initiation on complementary feeding [31].

##### Maternal and paternal factors

Three studies assessed maternal and paternal education as factors related to stunting in flooded areas [25, 31, 33]. One of the studies [25] observed no significant effects of paternal and maternal education on stunting two months post flood exposure. However, in the second study conducted a year after the floods [33], paternal education above primary level (middle, high school and college)was protective against stunting in children from flooded communities (*p* < 0.001). The third study [31] found that children in families with literate parents had a lower prevalence of stunting than those whose parents were illiterate or had only one literate parent (*p* = 0.0051). In the short-term following floods, non-significant effects of maternal and paternal age were reported. Del Ninno et al. [25] observed non-significant effects of maternal and paternal age two months after the floods. On the other hand, in the long-term period following floods, maternal age was noted as an important factor, while, for paternal age, non-significant effects were observed. Goudet et al. [28] noted maternal age as a predictor of stunting a year after exposure to floods. In another one year follow-up study [33], protective effects of paternal age at birth of the selected child (PR = 0.964, 95% CI: 0.917–1.013) were observed, but the effects were non-significant. Other maternal determinants of stunting identified by the studies included maternal BMI (OR = 4.45, 95% CI: 1.04–18.94) [28] and maternal height [25].

##### Household and socioeconomic factors

Four studies [14, 25, 31, 33] assessed household factors and their association with stunting in flood-affected areas. Overall, household factors, including the number of children under 5 years and household size, were not associated with stunting in the short-term and long-term following floods. In one study, stunting was not associated with the number (one or more than 2 in a household)of children under five years [14]. Similarly, in another study [32], no significant association was observed between the number of children below 5 years eating from the same kitchen and stunting in flooded areas. The third study observed no significant association between the increasing number of family members and stunting (*p* = 0.0844) [31]. The fourth study observed non-significant effects of household size on stunting in villages exposed to floods [25]. Assessed socioeconomic factors included socioeconomic class, per capita annual income and land ownership. In the long-term, socioeconomic status was associated with stunting, while per capita yearly income revealed protective effects. A more than two-fold increase (PR = 2.237, 95% CI: 1.128- 4.436) in the prevalence of stunting was noted among children from the scheduled caste (i.e., most deprived caste or social group in the country) [33]. Similarly, Islam et al. [31] reported socioeconomic class as a significant factor for stunting (*p* = 0.0113). An increase in per capita annual income by 1000 rupees was associated with a nearly 6% decrease in the prevalence of stunting for children from flooded areas (PR = 0.941, 95% CI: 0.894- 0.991) [33]. On the other hand, land ownership revealed non-significant effects on stunting one year after the floods (PR = 0.614, 95% CI: 0.366- 1.031) [33].

#### Underweight

##### Demographic factors (Child age and gender)

Four [14, 23, 24, 31] of the 14 studies assessed age as a determinant for underweight. Three of the four studies reported age as a risk factor for being underweight [23, 24, 31]. There were variations in the age groups more likely to be underweight, with studies reporting those aged 12–17 months [24], above 12 months [23] and those aged 48–60 months [31]. On the contrary, one study reported a non-significant difference in underweight status for the different age groups [14]. Three studies assessed [23, 24, 31] gender as a determinant of underweight. Overall, females in flood-affected areas were more vulnerable to underweight than males. In Bangladesh, females were more likely to be underweight than males [23, 24], but a study in India reported a significantly higher prevalence of underweight in males than females [31].

##### Child health-related factors and IYCF practices

Four studies [14, 23, 24, 28] identified child health-related factors as risk factors for being underweight in flood affected areas. The reported vulnerabilities included illnesses during the last two weeks preceding the study (6 months post-flood) [24], diarrhoea in the past two weeks preceding the survey [23], child birthweight [14], pre-existing underweight status (2 months post-flood) [28] and lack of vitamin A supplementation before the floods [24]. IYCF practices [31], including feeding of colostrum, no pre-lacteal feeds and exclusive breastfeeding, were associated with a lower prevalence of underweight. On the other hand, early introduction of complementary feeds (4–6 months) was associated with a higher prevalence of underweight [31].

##### Maternal and paternal factors

Three studies [23, 24, 31] identified maternal education as a determinant of being underweight. Maternal education was associated with being underweight three months after the floods (*p* = 0.01) [23]. In the six months following the floods, there was a significant difference in the prevalence of underweight pre and post-floods for children whose mothers had not gone to school (i.e. 0 years of education) (*p* < 0.001) [24]. In the flood-prone areas of Assam—India, a lower prevalence of underweight was observed among children with literate parents compared to those whose parents were illiterate or had one literate parent (*p* = 0.0026) [31]. Goudet et al. [28] reported maternal BMI as a predictor (OR = 3.51, 95% CI:1.02–12.05) of underweight status in children, a year after the floods.

##### Household and socioeconomic factors

Household factors, including child food share, family size and the number of children below 5 years, were associated with being underweight. An increase in family members was associated with being underweight (*p* = 0.007) [31]. The number of children under 5 years of age (i.e. ≥ 2) was significantly associated with being underweight one month after the floods (OR = 1.69 95% CI: 1.05- 2.72) [14]. Child food share revealed protective effects (OR = 0.92 95% CI: 0.86–0.98) on child underweight status a year after the floods.

Socioeconomic status and land ownership were related to being underweight following exposure to floods. Choudhury and Bhuiya [24] observed a double risk of underweight (OR = 2.01 *p* < 0.05, no confidence intervals reported) for children from low socioeconomic status families compared to other categories six months after the floods. Land ownership [23] was associated with underweight (*p* = 0.01) three months after the floods, with a lower WFA mean for those with no land ownership compared to those who owned land.

## Discussion

### Floods and undernutrition

Overall, stunting was the most commonly reported significant form of undernutrition in flood-affected areas. In addition to stunting, this review demonstrates the effect of floods on wasting and underweight among children under the age of five years. Effects of floods on nutrition have been highlighted in previous studies [39, 40, 41]. Existing evidence suggests that climate and weather extremes, including floods, are significantly associated with undernutrition in children [18, 19]. There was generally little evidence on the effect of floods on micronutrient deficiencies for children under the age of five years. The only assessed micronutrient deficiencies were vitamin A, D and iron deficiency anaemia. It was difficult to draw definite conclusions on their association with flooding in the affected areas as not much information was provided by the studies.

#### Undernutrition in flood affected areas

This review highlights the effect of seasonal and/or recurrent floods on undernutrition in children. A majority of the studies were from South Asian countries (Bangladesh, India, and Pakistan), where floods mostly transpired during the monsoon season, with some studies mentioning severe, early, prolonged and recurrent flooding. Over the past decades, the Centre for Research on the Epidemiology of Disasters (CRED) has reported a steady increase in floods [42]. Existing literature demonstrates constant variability in the occurrence of floods during the monsoon season [43]. In addition, effects of climate change predominantly in Asian countries have been suggested as the likely cause of variability in the monsoon floods in terms of intensity, frequency and duration [43]. In the seasonally and/or recurrently flooded areas, stunting was the most frequently reported significant form of child undernutrition with effects observed both in short-term and long-period following the floods. The studies also reported effects on wasting and underweight. Observed effects on stunting may be due to the long-term effects of the floods that are sometimes severe and recurrent as noted in some of the studies [27, 28, 32, 35]. No significant effects were reported in the other flood-affected areas other than the seasonally and/or recurrently flooded areas. Observations in these areas may be explained by limitations of the study design as all of these studies were of a cross-sectional design that is inherently unable to establish causality due to lack of time separating exposure and outcome measurements. Inconsistent with findings from the other flood affected areas, one study reported protective effects on stunting. However, this study reported the possible use of unreliable outcome data in relation to households’ exposure to flooding, thus liable to the observation of indefinite effects.

#### Short-term period

Stunting was the most frequently reported significant form of undernutrition in the short-term period following floods. However, both studies were rated as “Poor” quality, with the main weakness noted as a lack of sufficient time for observation of precise effects on the outcome. It is unlikely that significant effects on stunting would be observed within such a short time following exposure. Therefore, the observed effects could be explained by the nature and/or level of flood exposure in the study areas, probably due to recurrent and seasonal floods (sometimes severe) leading to long-term nutrition effects among children. For instance, in one of the studies [14] that mentioned recurrent floods, the observed effects may have been due to a flood that occurred two years before the study.

#### Long-term period

Stunting was the most frequently reported significant form of undernutrition in the long-term period following the floods. The findings of this review coincide with existing literature that suggests stunting is indicative of long-term nutritional consequences on the individual or population [35]. On the other hand, inconsistent findings were noted for both wasting and underweight.

### Factors associated with child undernutrition

#### Demographic factors (Age and gender)

This review suggests an overall increased risk for underweight and stunting among children above 12 months, while those below 24 months were more vulnerable to wasting. For instance, in the case of stunting, most studies reported an increased risk in older children (> 24 months). These observed effects may be due to increased overall exposure to floods among older children, especially in recurrently flooded areas. Stunting in younger children (< 24 months) is more likely to increase the risk of growth faltering and more so lead to negative short-term and long-term consequences of undernutrition [44]. Females in flood-affected areas were found to be more vulnerable to underweight, while male children were at a higher risk of both stunting and wasting. Findings of males being at a higher risk of stunting than females have previously been reported [35, 45–47]. In addition, societal and household-level preference for male children may partly explain why females are more likely to be underweight following floods.

#### Child health-related factors and IYCF practices

Besides demographic factors, diarrhoea was noted as the most frequently reported significant risk factor for undernutrition of all child health-associated vulnerabilities. Diarrhoea was observed as a significant factor for wasting and underweight among children in the short-term following floods, while for stunting, observed effects more than a year later (15 months) were related to the duration of diarrhoea during the floods. The occurrence of diarrhoea in the period following floods could probably be due to contaminated water sources. In developing countries, exacerbation of diarrhoeal diseases due to compromised sanitation and water supply following floods has previously been reported [48–51]. Existing evidence reports an increased risk of stunting for every episode of diarrhoea [44]. The review findings, therefore, demonstrate the importance of sustained Water, Sanitation and Hygiene (WASH) practices before, during and in the period following floods. Other vulnerabilities included pre-existing undernutrition status before floods (underweight and stunting), child illnesses, duration of ARI during the floods, child birthweight and vitamin A supplementation status before the floods. The review also highlights the importance of optimal recommended IYCF practices, i.e., exclusive breastfeeding and initiation of complementary feeding at 6 months, as they were associated with a lower prevalence of all three forms of undernutrition (wasting, stunting and underweight) in flood-prone areas.

#### Maternal and paternal factors

Overall findings of the review suggest no significant effects of maternal education on undernutrition within the four months (short-term)following floods, as reported in three [23, 25, 26] of the four studies conducted within this timeframe. The absence of effects was especially reported for wasting, a form of undernutrition due to short-term nutritional consequences. On the other hand, maternal education seemed to be an important factor in the long-term period following floods, with significant effects observed for underweight. Similarly, the review noted the protective effects of paternal education on both wasting and stunting in the long-term period following floods. Presumably, the observed effects of both maternal and paternal education in the long-term or recovery period following floods may be due to better knowledge of nutrition-related practices, higher incomes or savings and the ability to invest in better and long-term coping strategies or income-generating activities. On the other hand, during the immediate and short-term period, the effects of floods may be too difficult and destructive to cope with, even for educated mothers and fathers. For instance, this study noted that households that lost assets dedicated to agricultural activities and those that depended on their food stocks had their children more vulnerable to wasting [26, 33]. It is plausible that with the available resources and assets depleted or lost during and in the period following floods, immediate (e.g., disease and inadequate dietary intake)and underlying causes (e.g., food insecurity and inadequate care and child feeding practices) of undernutrition begin to emerge thus increasing children’s vulnerability to acute undernutrition. Other maternal factors associated with undernutrition outcomes included BMI (underweight), age at first delivery, age at birth of child and height. Maternal age at first delivery and birth of the child revealed protective effects against stunting and wasting, respectively. Protective effects of maternal age on stunting have previously been reported [52, 53]. Maternal BMI (underweight) was a predictor of stunting and underweight a year after the floods [28]. This suggests that an underweight child in the period following floods is more likely to have had an underweight mother after the floods. Underweight mothers are more likely to give birth to low birth weight (LBW) babies due to Intra Uterine Growth Restriction (IUGR) [44]. Therefore, significant effects of maternal BMI demonstrate the impact of maternal underweight status on child nutrition status in the long-term period following flood exposure. Observed impacts on stunting may indicate an intergenerational cycle of malnutrition in flood-affected areas, particularly relating to mothers' poor nutrition status (Low BMI) before and during pregnancy, increasing the risk for IUGR-low birth weight [44, 54]. IUGR-low birth weight has been linked to child nutrition status, with LBW as a strong risk factor for stunting [55, 56]. Therefore, in areas particularly affected by recurrent floods, the chances are that children born with LBW may grow into stunted children. LBW babies have increased vulnerability to common childhood diseases, which are immediate causes of undernutrition [57] and a risk factor for underweight [47, 58].

#### Household and socioeconomic factors

Family size (number of family members) was associated with wasting and underweight. These findings perhaps relate to issues of food security, child food share and care practices [50, 59]. Particularly for children from larger families, it may be that they experience inadequate food intake due to the compromised ability of their families to meet food needs after the floods. On the other hand, stunting was not associated with household size, the number of family members (family size) and children under five years. The observed non-significant effects of the household-related factors on stunting may well be attributed to the innate cross-sectional study design. Socioeconomic status was associated with all three forms of undernutrition (i.e., stunting, wasting and underweight) in flooded areas. Undernutrition was found to be common among children from low socioeconomic households. Previous studies have reported similar findings [34, 60–63].

Land ownership was significantly associated with wasting and underweight three months after the floods, with a lower WFA mean for those without land ownership (landless). On the other hand, there were no observed effects of land ownership on stunting and wasting one year following the floods. The observed non-significant effects one year later may presumably be attributed to the inability of the cross-sectional study design to establish causality. It may also be that, even for people with land ownership, their crops have been washed away by floods, and thus are unable to provide enough food for their families. Stewart et al. [23] confirms this view, with significant loss of crops, livestock and house damage observed for families heavily affected by the floods. Overall, these findings suggest that all flooded households need to be supported in the immediate and short-term following floods regardless of whether they own or do not own land.

### Methodological quality, limitations and strengths of included studies

Overall, the quality of the evidence was fairly weak as the majority of the included studies were rated as “Fair”. Therefore, the review remains inconclusive on the findings based on the observed limitations and thus should be interpreted cautiously. There were several difficulties encountered while reviewing the current evidence. One key limitation was that; all studies were of a cross-sectional design, which is innately incapable of investigating causality mainly due to temporal relationship drawbacks. Although, in the majority of the studies, the outcome was measured in the post-flood period, it would likely be challenging to ascertain whether the observed effects occurred before or after the flood event or were due to other environmental factors or catastrophic events. For instance, of the 14 studies, only 8 controlled for confounders but not all the relevant or possible confounders were considered. Other limitations of the review included the following: Inconsistent findings within and across studies presumably may have been due to intrinsic differences. Studies varied by age group, sample size, measure and definition of the primary exposure (floods), as well as duration of follow-up (timeframe between exposure and measurement of outcomes), making it difficult to effectively synthesise and draw strong conclusions from some of the study findings. For instance, stunting was noted as the most frequently reported significant form of undernutrition in the short-term, while the findings were inconsistent for wasting and underweight. The review could not draw a strong conclusion on the most significant form of undernutrition in the short-term period as the observed effects on stunting were highly unlikely due to the limited timeframe between exposure and measure of the outcome. Secondly, most studies lacked clarity on vital information relating to floods including type, frequency, duration, magnitude and level of exposure. Presumably, inadequate information on flood variables could mean discrepancies in the impact of floods on nutrition outcomes, which the study could not ascertain. Furthermore, retrospective data collection may have introduced reporting bias leading to underestimation of the effect sizes for the different nutrition outcomes. The use of secondary data in 7 of the 14 studies may have introduced bias in each of the studies that the review could not ascertain. Finally, using NCHS median reference standards in six of the 14 studies may have limited the ability to draw valid links between floods and undernutrition outcomes, as the WHO Child growth reference standards replaced these due to some limitations [64].

#### Study limitations and strengths

Studies obtained were essentially from LMICs in South Asia countries where floods mainly occurred during the monsoon season. This limits the generalisability of the review, as findings may not apply to other LMICs, for example, countries in Sub-Saharan Africa where flooding might be due to other causes. Flood occurrences in Africa and South America have been linked to the natural El-Nino phenomenon or climate change [65]. Similarly, because most of the studies were from rural settings, findings of this review may not apply to urban areas. Urban areas too are affected by floods, but it seems existing research has focused primarily on rural areas. Although this may probably be due to the increased vulnerability of people from rural settings, it is vital that the impact of floods in urban settings, for example, slums or peri-urban areas is also explored. Secondly, this review included the search of articles from mainly international databases. Due to the nature of countries considered (LMICs), it is plausible that some relevant articles were missed as they either have not been published or appear in other databases that were not searched (publication bias). Furthermore, the exclusion criteria may have introduced some selection bias. For instance, because the search strategy focused on only English language articles, likely, other relevant articles may have been excluded. On the other hand, the strengths of the review included the use of the WHO Child growth reference standards for most of the studies (8 of 14), which provided high precision to the review findings. Secondly, 7 of the 14 studies used primary data demonstrating that for each of the studies, data were collected to answer definite study objectives on the impact of floods undernutrition. In addition, eight of the 14 studies adjusted for potential confounders, which may have enabled observation of precise effects, despite individual study weaknesses. Finally, the study comprehensively reviewed existing evidence on the impact of floods on child undernutrition outcomes while considering the duration of the studies post-flood exposure and associated factors.

### Suggestions for future research, policy and practice

One observation was that there is scant evidence on the impact of floods on undernutrition in other LMICs across the globe except for a few countries in South Asia, including India, Bangladesh and Pakistan. Over the past two decades, other LMICs in Asia, Africa and South America have been affected by floods. However, it appears that not much has been done to ascertain their impact on child undernutrition outcomes. Therefore, more research is recommended to document evidence from other LMICs across the globe and in urban settings. Research funding is also needed to conduct this research in these countries. The type of floods was not clearly stated in most of the studies. The EM-DAT disaster profiles demonstrate that riverine floods affect more people than flash and coastal floods. Although this review identifies floods as mainly occurring during the monsoon season in South Asian countries, it is unclear which type of flood impacts undernutrition. Therefore, to draw more robust conclusions on the impact of floods on undernutrition, it is important that future studies clearly state the specific type of flood, frequency, duration and magnitude. To enable a broad investigation, future research should focus on assessing all forms of undernutrition, including micronutrient deficiencies and utilise other methods of nutrition assessment other than anthropometric measures (clinical and biochemical methods). In addition, obtaining robust evidence will require assessing all important determinants of undernutrition (i.e., in flood-affected areas) as identified by previous studies, adjusting for all-important confounders, considering a control group and, if possible, conducting large prospective studies. Prospective studies could be initiated before, in the immediate or short-term period following floods to identify more objective causal pathways of undernutrition in flood-affected areas, including the associated mediators and moderators. Considering the effect of diarrhoea, as noted in this review, there is a need for future research investigating its association with undernutrition before and after floods, as well as the efficacy of possible intervention strategies (e.g., prophylactic antibiotics and/or health education focusing on hand sanitation). Further investigation is needed to provide strong evidence of the significance of maternal and paternal education and IYCF practices as determinants of undernutrition in flood-affected areas, especially in the short-term following floods.

#### Policy and practice

Based on the study findings, this review highlights various recommendations and suggests ways towards response, alleviation, and prevention of undernutrition in the short-term, intermediate and long-term periods following floods. To prevent and reduce the risk of wasting in flooded areas, emergency and relief organisations need to target nutrition support and response for children with increased vulnerabilities. Male children need to be closely monitored during the immediate and short-term period following floods (1-4 months), as they are more vulnerable to acute and chronic undernutrition. However, female children also need to be monitored as they are more likely to be underweight than males following floods. In addition, the nutrition status of younger children (0–24 months) needs to be monitored during and in the period following floods to avoid wasting, long-term nutritional impacts and associated consequences. In the six months and one-year following floods, nutrition support needs to be targeted for children with pre-existing undernutrition, underweight mothers, and those from larger families and low socioeconomic households as they are at a higher risk of undernutrition. The increased risk of undernutrition among children with pre-existing undernutrition status or underweight mothers validates the importance of nutrition surveillance in the immediate post-flood period, to identify children most at risk of undernutrition and to guide better response and prevention measures. Recommended is the strengthening of girl-child education policies, especially in rural areas, and implementation of nutrition education programs aimed at promoting IYCF and WASH practices during and after floods, mainly targeting girls and women of reproductive age. Designing a Social and Behaviour Change communication strategy may also help promote the uptake of recommended nutrition behaviours. Finally, interventions in flood-affected areas need to focus on preventing and reducing child stunting levels and its long-term consequences.

## Conclusions

This study provides some evidence of the impact of floods on undernutrition, mainly in the forms of stunting, wasting and underweight in children under five years in LMICs. The review remains inconclusive on the effect of floods on micronutrient deficiencies. Overall, this study identified stunting as the most common form of undernutrition in flood-affected areas besides wasting and underweight. In view of undernutrition during the post-flood period, the review is inconclusive on the most significant forms within the short-term and intermediate periods following floods due to weak and limited evidence, respectively. In the long-term period, stunting was noted as the most significant form of undernutrition, while inconsistent findings were noted for both wasting and underweight. The quality of the evidence was fairly weak, with the main challenge lying in the inability of the studies to establish causal pathways for the observed effects, as all the studies were of cross-sectional study design. Future research utilising long-term prospective data (longitudinal studies) is indispensable to provide more robust evidence to guide better prevention measures, response decisions and short-term and long-term interventions in flood-affected and/or prone areas. With the projected increase in weather variability and climate change, more severe (seasonal) and recurrent floods may be expected. Consequently, clear plans and strategies for preventing and reducing the long-term impact of floods on child undernutrition need to be drawn. Programmes in flood-affected areas need to integrate nutrition approaches and indicators as part of flood preparedness, Early Warning and surveillance systems and consider a multi-sectoral approach toward child undernutrition.

## Supplementary Information

**Additional file 1. **Quality Assessment of studies performed using the NIH tool for Observational Cohort and Cross-Sectional Studies.

## Data Availability

Data sharing does not apply to this article as no datasets were generated or analysed during the current study.

## Abbreviations

ARI: Acute Respiratory Infection
BMI: Body mass index
CRED: Centre for Research on the Epidemiology of Disasters
HFA: Height-for-age
IPCC: Intergovernmental Panel on Climate Change
IUGR: Intrauterine growth restriction
IYCF: Infant and Young Child Feeding
LMICs: Low- and Middle- Income-Countries
NCHS: National Centre for Health Statistics
SDG: Sustainable Development Goal
UNICEF: United Nations Children's Fund
WASH: Water, Sanitation and Hygiene
WFA: Weight-for-age
WFH: Weight-for-height
WHO: World Health Organization
