# Supplementary material for: Impact of floods on undernutrition among children under five years of age in low- and middle-income countries: a systematic review
Source: Environ Health. 2022 Oct 24;21:98. doi: 10.1186/s12940-022-00910-7 (PMC9590165; doi:10.1186/s12940-022-00910-7)
Supplement: Supplementary file 1 — Additional file 1. Quality Assessment of studies performed using the NIH tool for Observational Cohort and Cross-Sectional Studies. [file 12940_2022_910_MOESM1_ESM.docx]

**Supplementary Information**

**Additional file 1: Quality Assessment of studies performed using the NIH tool for Observational Cohort and Cross-Sectional Studies**

| **No.** | **Reference (Author and year)** | **Stewart et al., 1990** | **Choudhury and Bhuiya, 1993** | **del Ninno C et al., 2001** | **Hossain& Kolsteren, 2003** | **del Ninno & Lundberg, 2005** | **Goudet et al., 2011** | **Rodriguez-Llanes et al., 2011** | **Hossain et al., 2013** | **Quddus and Bauer, 2013** | **Islam et al., 2014** | **Rodriguez-Llanes et al., 2016a** | **Rodriguez-Llanes et al., 2016b** | **Gaire et al., 2016** | **Dimitrova and Bora, 2020** |
| --- | --- | --- | --- | --- | --- | --- | --- | --- | --- | --- | --- | --- | --- | --- | --- |
| **1.** | **Research question defined** | Y | Y | Y | Y | Y | Y | Y | Y | Y | Y | Y | Y | Y | Y |
| **2.** | **Study population clearly specified and defined?** | Y | Y | Y | Y | Y | Y | Y | Y | Y | Y | Y | Y | Y | Y |
| **3.** | **Participation rate of eligible persons at least 50%?** | Y | Y | Y | Y | Y | Y | Y | Y | Y | Y | Y | Y | Y | Y |
| **4.** | **Groups recruited from the same population and uniform eligibility criteria** | Y | Y | Y | N | Y | Y | Y | Y | Y | Y | Y | Y | Y | Y |
| **5.** | **Sample size justification** | N | Y | Y | N | Y | Y | Y | Y | Y | Y | Y | Y | Y | Y |
| **6.** | **Exposure assessed prior to outcome measurement** | N | Y | Y | Y | Y | Y | N | N | N | N | Y | Y | Y | Y |
| **7.** | **Sufficient timeframe to see an effect** | N | Y | N | Y | Y | Y | N | N | CD | CD | Y | Y | Y | Y |
| **8.** | **Different levels of the exposure of interest** | N | N | Y | N | Y | Y | NA | N | N | N | NA | NA | NA | N |
| **9.** | **Exposure measures and assessment** | N | Y | Y | N | Y | Y | Y | Y | N | N | Y | Y | Y | Y |
| **10.** | **Repeated exposure assessment** | NA | NA | NA | NA | NA | NA | NA | NA | NA | NA | NA | NA | NA | NA |
| **11.** | **Outcome measures (accurate and reliable)** | N | N | N | N | N | N | Y | Y | Y | Y | Y | Y | N | Y |
| **12.** | **Blinding of outcome assessors** | N | N | Y | N | Y | Y | N | N | N | N | Y | Y | Y | Y |
| **13.** | **Follow up rate** | Y | Y | Y | N | Y | Y | Y | Y | Y | Y | Y | N | Y | Y |
| **14.** | **Statistical analyses (key potential confounding variables adjusted for)** | Y | Y | N | N | N | N | Y | N | N | N | Y | Y | Y | Y |
|  | Quality Rating (Good, Fair, or Poor) | **Poor** | **Fair** | **Fair** | **Poor** | **Fair** | **Fair** | **Fair** | **Fair** | **Poor** | **Poor** | **Good** | **Good** | **Fair** | **Good** |
| **N, no; Y, yes; NA, not applicable; CD, cannot determine** | | | | | | | | | | | | | | | |
